# The Outlook of the Development of Innovative Products from Biocompatible Natural Spider Silk in the Beauty Thread-Lifting Industry

**DOI:** 10.1007/s13659-020-00291-9

**Published:** 2021-01-04

**Authors:** Chen Qing, Qi-yan Li, Nan-nan Xue, Shi-meng Yuan, Chuan-jun Liu, Cheng-gui Zhang, He-wei Li, Yu Zhao

**Affiliations:** 1 Yunnan Provincial Key Laboratory of Entomological Biopharmaceutical R&D, Dali University, Dali, 671000 People’s Republic of China; 2 Yunnan National-Local Joint Engineering Research Center of Entomoceutics, Dali University, Dali, 671000 People’s Republic of China; 3 Center of Stomatology, The First People’s Hospital of Yunnan Province, Kunming, 650032 People’s Republic of China; 4 Jiangsu Weibo Hi-Tech Biological Technology Co., Ltd., Changzhou, 213000 People’s Republic of China

**Keywords:** Thread lift, Face rejuvenation, Barbed suture, Natural spider silk, Review

## Abstract

**Abstract:**

Embedding thread lift rhytidectomy, also known as “thread lifting” in China, with the natures of simple operation, less trauma and quick recovery, is progressively used in clinical practice as a new technology of face lifting. Herewith, a brief introduction of the previous advances of thread lifting techniques and materials in the facial beauty industry, combined with the discussion on various types of sutures, common complications, and the site of actions were provided. The main limitations of present thread lifting material include: (1) the use of non-absorbable sutures is liable to cause allergies and a series of complications; (2) the absorbable sutures are easily degradation, and people need to reshape in a relatively short period. Therefore, the high biocompatible spider silk was proposed as a novel material of thread lifting suture and related devices, the advantages and preliminary achievements on spider silk were also addressed.

**Graphic Abstract:**

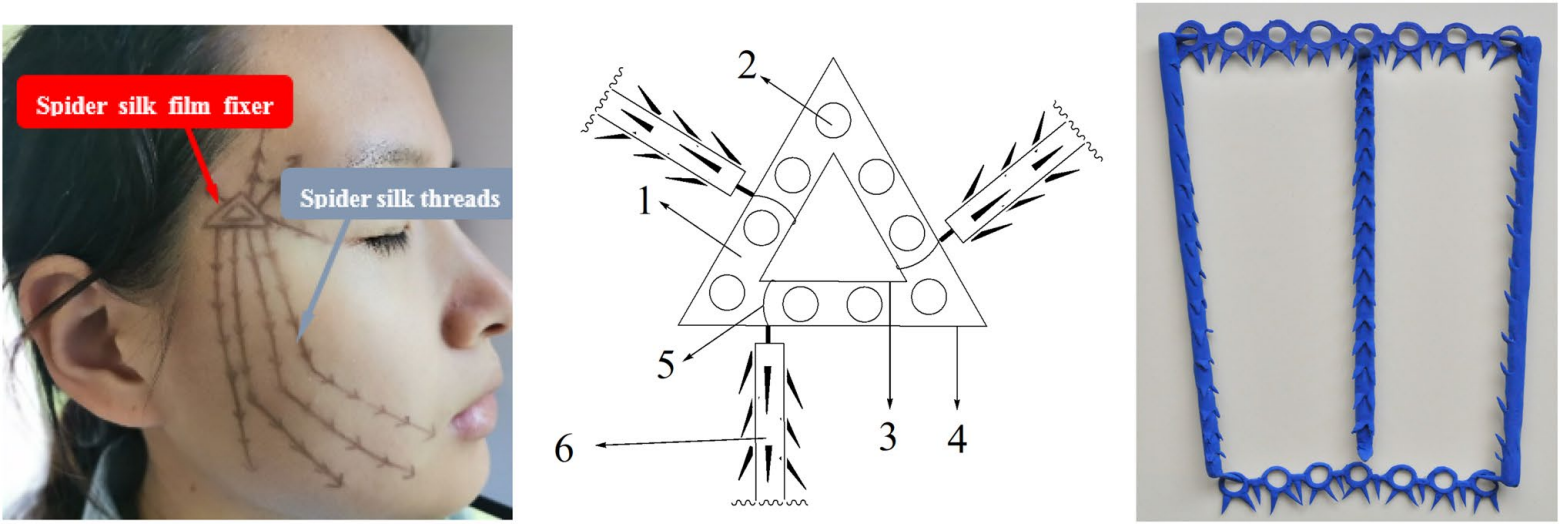

## Introduction

Since the modern facial lifts were first described by the German Surgeons in 1910 [1], there has been a substantial amount of plastic surgeries being performed around the world. According to International Society of Aesthetic Plastic surgery (ISAPS) reports, five countries comprised the United States, Brazil, Japan, Mexico and Germany have the highest rates of cosmetic (invasive and non-invasive) surgeries that account for 41.1% of the world [2]. China as a country with a large population, rapid economic development, and continuous improvement of people’s living standards, especially with an increasing aging population, the development of a green, pollution-free, and lower-risk medical beauty industry met greater urgency and importance. People (especially women) have more and more anti-aging beauty needs on improving facial relaxation, flabby breasts, and sagging buttocks. The chasing a good-looking image and concerning for health have cultivated the medical cosmetology becoming one the hottest branches in the tertiary industry.

The face is one of the most complex areas in the human body. Bone, ligaments, muscles, fat, and skin are the critical factors in the layered arrangement of the face [3]. In addition, the face is also a principal focus of the medical aesthetics research community and plastic surgeons, and appropriate facial improvement can positively affect social interactions. With aging processes, our facial soft tissues would change and become loose. The skin thins and loses elasticity; lipoatrophy and gravity are mainly causations for the observed age-related facial feature.

With the in-depth understanding of the facial anatomy and aging process, the significant improvement of surgical techniques and treatment strategies, facial beauty has developed not only in quantity but also in quality [4]. According to the American Society of Aesthetic Plastic Surgery (ASAPS), traditional surgical cosmetic procedures such as rhytidectomy, are still quite popular. However, surgical plastic surgery is rather aggressive, with long postoperative recovery time, there are also common complications such as scars, skin flap necrosis, infection, hematoma, seroma, greater auricular nerve injury and facial nerve injury, ear deformity, parotid duct injury, hair loss, poor aesthetic outcome, infection and deep vein thrombosis, as well as the risks associated with general anesthesia and even conscious sedation [5, 6].

With the aesthetic industry floods into the market, surgeons and patients are looking for less invasive technologies, fewer complications, slightly painful, convenient for plastic surgery applications, and faster recovery of physiological functions. Therefore, most young patients prefer moderate cosmetic improvement (such as minimally invasive surgery) to reduce the complication rate and increase the possibility of quick healing [6, 7]. Based on the surgical and non-surgical rejuvenation plastic data released by ASAPS, from 2014 to 2018, the proportion of facial plastic surgery has fallen by 21% in 5 years, while the growth rate for non-surgical facial beauty was 50.2%, this further supported the necessity and trend of searching for non-surgical or less invasive methods [8].

Minimally invasive cosmetology is the most dynamic field of contemporary medical cosmetology [9]. The data of ASAPS indicated that the injection means were used to fill or shape the face with other intermediate treatments such as hyaluronic acid (HA) and botox, accounting for 80.9% of less-invasive procedures at the end up to 2019 [10], following by laser treatment, radiofrequency ablation treatment, chemical peeling and other skin surface treatment tools. Recently introduced the quick-recovery and small-incision face lifting, micro-focused ultrasound with high-resolution ultrasound visualization (MFU-V), and internal suture suspension that can surmount obvious obstacles of traditional surgical facelift [11]. The common advantages of this type of less-invasive plastic surgery include fast operation time, no general anesthesia, short recovery time, and fewer complications, which brings a lot of convenience to patients and surgeons [12]. However, it is worth noting that the use of fillers may cause stiff countenance and unnatural contours after the face is filled. Resurfacing technologies such as laser treatment and MFU-V can improve the skin surface, but they cannot adequately address the need for sagging soft tissue, which is an essential step in reshaping youthful face [13]. As a representative of micro plastic surgery in the new era, thread lifting, also known as suture suspension, has gradually occupied the stage in facial lifting. The development of the emerging field of thread-lift has brought new exploration directions to the increasingly fashionable beauty industry. Furthermore, focusing on exploring the sources of sutures, as well as selecting of innovative materials will be important targets in future research.

## The Development of Thread Lifting Technology

During thread lifting operation, the skin is lifted by subcutaneous implantation of collagen thread to trim wrinkles and laxity. As a novel cosmetic project of rejuvenation, this skill becomes rapidly popular in developed countries such as Japan, South Korea, European and North American countries. The first facial suspension operation with barbed sutures was performed by Sulamanidze and his colleagues in the 1990s, and then it has become popular among dermatologists, plastic surgery and other cosmetic surgeons [14]. In addition to its main applications in facial rejuvenation, the thread lifting is also involved in body remodeling such as buttocks lift, breast lift, arm lift, abdominoplasty, vaginal tightening and uterine suspension and other branches [15–20]. The operational positions of face-lift are based on the upper, middle and lower face, whilst the major treatment parts consisted of forehead wrinkles, frown lines, and eyebrow lifts on the upper face, fat sagging in the zygomatic area, ptosis, nasal tip, nasolabial folds, bucco-mentonian grooves of the middle face, double chin, marionette lines and mandibular of the lower face, including the neck lines, etc. [7, 21–23]. This new type of beauty project can generate nearly tens of billions of economic benefits every year. As a vital integral part of the thread lifting, the industry leaders of thread lifting suture in technology and materials are United States, Japan, and South Korea. It could be seen that the level of R&D and industrialization of domestic related high-end medical aesthetic materials companies in china still has huge room for development compared with foreign countries.

### Type of Sutures for Thread Lifting

Thread lifting sutures are usually made of monofilament suture material. Original smooth sutures made of polymer materials such as polypropylene, polylactic and polytetrafluoroethylene possess significant shortcomings such as difficulty in fixing, sliding, and instability, which causes the permanent fixation effect in the body is unsatisfied [24, 25]. In 1964, Alcamo took the lead in proposing the concept of barbed suture [26]. The purpose was to close wounds without tying a knot, but it was unexpectedly used in esthetic applications. With the emergence of polypropylene barbed sutures, the load-bearing capacity of facial sutures has been greatly improved [27].

Barbed suture is a special type of suture thread, which is characterized by many sharp fine teeth distributed at an acute angle on the surface of the thread [28]. The suture material can be categorized as non-absorbable and absorbable (Table 1) [11, 14, 25, 28, 29, 33, 39, 42]. Most of the non-absorbable threads are made of polypropylene, such as the Aptos thread proposed by Sulamanidze and colleagues in 2002, which was the first commercialized suture with multidirectional barbs [14]; the Contour line was approved by the FDA in 2004 as a thread lifting suture with unidirectional barbs for rejuvenating the middle and lower parts of the face [6, 11, 25]. While the absorbable thread can be made from polydioxanone (PDO), for example, the Quill Knotless Tissue-Closure Device and Synthetic Absorbable Barbed Suture are FDA-approved absorbable barbed PDO threads for soft tissue closure [11, 28]. In addition, the materials of absorbable suture using other polymer materials such as polyglyconate, polylactic acid-glycolic acid copolymer (PLGA), poly-_*L*_-lactic acid (PLLA), and poly *p*-dioxanone (PPDO) [39]. The Silhouette Instalift is a type of PLLA suture with bidirectional PLGA copolymer cones, which was approved by the FDA for temporary intermediate suspension for the subcutaneous bumps of the cheek, this type of thread can last up to 12–18 months in the body [11].

**Table 1:** Summary of barbed suture characteristics

Suture material	Trade name	Producer	Placement	References
Nonabsorbable threads				
polypropylene suture	Aptos thread	Aptos, Ltd., Moscow, RUS Kolster methods, Inc., Anaheim, CA	Free-float	Sulamanidze et al. [14] Paul et al. [28]
	Contour thread	Surgical Specialties, Corp., Reading, PA	Anchor	Kaminer et al. [6] Tong et al. [11] Gülbitti et al. [25]
	Isse Endo Progressive Facelift suture	Kolster methods, Inc., Anaheim, CA	Anchor	Tong et al. [11] Paul et al. [28]
Absorbable threads				
Polydioxanone suture (PDO)	Quill thread, Quill Knotless Tissue-Closure Device	Quill Medical, Inc., Research Triangle Park, NC Angiotech Pharmaceuticals, Inc., Vancouver, British Columbia, CAN	Anchor/Free-float	Tong et al. [11] Paul et al. [28]
	VOV-LIFT	GLK International, Seoul, Kr	Anchor/Free-float	Kang et al. [42]
polyglyconate	V-Loc 180	Covidien, New Haven, CT	Anchor/Free-float	Kwon et al. [29]
Poly-_*L*_-Lactic acid suture (PLLA)	Silhouette Instalift	Silhouette Lift, Inc., Westwood, MA	Anchor	Tong et al. [11]
	Silhouette soft	Sinclair Pharma, London, UK	Anchor	Gülbitti et al. [25] Ogilvie et al. [33]
Poly-*p*-dioxanone suture (PPDO)	Hengsheng PPDO thread	Tianjing Dongnan Hengsheng Inc., Tianjing, CN	Anchor	Tang et al. [39]

Moreover, in the light of the distribution direction of the suture, which are divided into barbed threads with unidirectional tension, multidirectional tension as well as bidirectional tension, and the connection between needle and thread of barbed suture is also different, they could be exhibited as styles such as needle tail belt thread, needle center belt thread, hollow needle belt thread, needle outer winding, etc. [28]. Furthermore, the textures of the surgical suture could be classified into two categories: floating-type thread and fixed-type thread, which the implantation methods by free-floating and anchoring separately (Table 1). Different surgical types have different options. For example, when considering the functional anatomy of the face from the perspective of facial expressions, a floating-type thread is generally preferred instead of fixed-type [29].

### Common Complications of Thread Lifting Beauty

Using a minimally invasive method to implant barbed suture, there are few contraindications except for the case of allergies to its materials. However, non-absorbable barbed sutures have the defects of high complications and high reforming rate [30, 31], and its complications include severe pain, skin dimpling, foreign body reaction, infection, thread protrusion, edema, facial asymmetry and other adverse reactions, and it is difficult to remove it from the face. Residual wire fragments may also cause adverse symptoms [25, 32, 33].

However, a large number of studies have shown that the use of absorbable PDO thread has fewer complications, and it can automatically degrade within 6–12 months in vivo [11]. Absorbable threads such as PDO and PLLA are considered to be stimulators of collagen and are believed to have long-term benefits for stimulating facial rejuvenation [34, 35]. Although the newly published methods show that the safety and effectiveness of the use of absorbable sutures have been significantly improved, most of the clinical observation times reported in the existing literature is shorter than one year, and there are no studies on those who have a follow-up time of more than 3 years [11], this is also the main shortcoming of absorbable barbed sutures. Nevertheless, the above facts imply that absorbable barbed suture such as PLLA are fully biodegradable, thus reducing the risk of infection or the need for stitches removal, as well as reducing the number of cavitations, extrusion or palpable adverse reactions [36].

### The Site of Action

The latest generation of barbed suture is available in both non-absorbable and absorbable materials, which possess a variety of lengths and different types of inlaid needles [37]. Different thread-anchoring positions and types are used for different manifestations, most of the thread inserted into the temple area, are mainly lifting Superficial Musculo-Aponeurotic System (SMAS) [38, 39]. Moreover, plastic surgeons found the safe implantation layer from the anatomical level after years of practice: SMAS superficial surface, forehead to the deep frontal muscle surface, temporal to the superficial temporal fascia surface [40]. However, it should be noted that the anchoring points should avoid blood vessels, utilizing Doppler ultrasound, for example, can avoid vascular complications by avoiding the anterior branch of the superficial temporal artery, located near the hairline and in the superficial temporal fascia layer [41].

The placement for suture suspension includes anchoring, viz. one end is stitched into a relatively fixed tissue along the subcutaneous plane of the face, such as periosteum and fascia; and the other is free floating to spread the barbed suture into the tissue and use the tension of suture to increase the tightness between tissues [28]. There are also reports where a suture thread is inserted vertically down between the anterior and inferior zygomatic areas, taking into account the specific characteristics of the anatomy of the bones between Asians and Caucasians [42]. The current methods for evaluating the effects of facial lifting are generally the 5-point Global Aesthetic Improvement Scale (GAIS), 3D imaging system and subjective satisfaction evaluation [29].

The above briefly outlines the current development of thread lift technology in the minimally invasive medical aesthetic industry. The absorbable/non-absorbable suture materials in the present market are mainly various polymer materials with single composition. Considering the advantages of high toughness and high biocompatibility of natural spider silk, the author's team (Yunnan Provincial Key Laboratory of Entomological Biopharmaceutical R&D) scheduled to apply this biomaterial to thread lifting cosmetic products, hoping to develop a new blue ocean of medical biomaterials.

## New Material for Barbed Suture—Natural Spider Silk

### Introduction of Natural Spider Silk

Spiders belong to Arthropoda, the Araneae of the Arachnoidea, most of them can spin silk to make a net, which have a strong ability of survival and adaptation [43]. At present, there are more than 40,000 species of spiders in the world, which are distributed all over the world except Antarctica. There are about 3,800 species of spiders recorded in China [44]. Spider silk is made of proteins secreted by their silk glands that condense when exposed to air. Spiders can synthesize spider silk throughout their life, and long-term biological evolution endows this biological protein polymeric fiber with special properties [45]. Most spiders secrete more than one kind of spider silk, and each spider silk has its specific function. It has been reported that one type of spider can produce at least 7 different kinds of silk, each with a different function [46]. At present, the most investigated silk-producing is the *Araneus ventricosus* which weaving a spherical web, whose main ampulla gland secretes the dragline silk [47]. The frame of dragline silk is the strongest among the seven kinds of spider silk, which is characterized by: (1) high strength, toughness, elasticity, tensile strength and other mechanical properties. The strength of dragline silk is five times that of steel, and three times that of aramid, which is known as “biological steel protein”; (2) it possesses simultaneously the properties of thermal stability to high temperature and low temperature resistance, as well as chemical stability; (3) excellent biocompatibility and biodegradability [48–51]. Due to the above-mentioned excellent characteristics, natural spider silk has a wide range of uses as new biological material. It is worth to be further investigated and developed to be better fiber materials, which could be applied in various fields.

### Application of Natural Spider Silk

Early as 1709 there were reports of using spider silk to weave stockings and gloves. During World War II, spider silk was applied to cross-hair and bulletproof vests on sights [52]. The further research demonstrates that spider silk can be used for parachute ropes, aircraft composite materials, and astronaut costume in the military [47]. Spider silk in industry can make high-strength structural materials, wheel tires, fishing nets, etc. [43]. Besides, spider silk has the potential ability to become bioengineering materials in medical and health care owes to its natural protein fiber and high biocompatibility with the human body, for instance, fibrous skeleton of heart, vascular stents, artificial joint ligaments and so on [53, 54]. They can also be in prospect of producing artificial tendons, blood vessels, bio-skin, hemostatic membranes, biofilms used in burn surgery and drug delivery carriers [55]. Furthermore, spider silk can also be utilized as a suture for surgical operations with the advantages of the high toughness and biodegradability [43, 49, 52]. Nevertheless, there is no substantial scientific evidence about using spider silk to make thread-lift sutures. In 1998, Bickel et al. reported a subcutaneous “spider silk” suture to address the thread easily break and discomfort of long incisions in patients, and there were no related complications within 10 years [56]. There is literature regarding the use of spider silk as an antibacterial coating on silk surgical sutures demonstrates to prevent bacterial attachment in the recent [57]. The overall reported of the above suggested the great benefits of natural spider silk as suture materials.

Current sutures are mostly consisted of absorbable high-molecular polymer, despite they can be decomposed into non-toxic or harmless substances in human tissues, there are obvious drawbacks with the texture is brittle, easy to break after oxidation, especially to fail after long-term storage, and difficult to shape. Therefore, the corresponding key common technical problem that the industry needs to solve is that the suture loses its efficacy rapidly due to oxidation and aging in the skin. Thus, patients with cosmetic thread lifting have to endure the inconvenience of re-implantation once every 1–2 years. Furthermore, the technical flaws worth mentioning lies in that the present thread-lift sutures generally can only be made into a straight line with intermittent barbs or cones without other shapes that result in the thread lifting cosmetic surgery method is single and the fancy thread lifting operations cannot be completed, making the aesthetic effect uniform and non-personal, thus induces the characteristic market imperfections. Hence, there is an urgent need in the market for a thread-lift beauty suture and related fixing devices that gather simultaneous “eight advantages” which means good biocompatibility, no rejection, better flexibility, hard to break, strong plasticity, uneasy to aging, unique shape and usage, and can be absorbed by biological organisms. The critical and common technical solutions in this market may bring tremendous and revolutionary vitality to the global beauty industry.

### Innovative Application of Natural Spider Silk on Barbed Sutures Beauty

Taking into account the limitations of existing absorbable/non-absorbable sutures, Yunnan Provincial Key Laboratory of Entomological Biopharmaceutical R&D is committed to developing an innovative product that combines natural spider silk and beauty barbed sutures. It is worth mentioning that although spider silk has broad application prospects in biotechnology, national defense, industry and medicine; spiders cannot be cultivated at a high density like silkworms due to their cannibalism [58], while the spider itself spins only a small amount, both above severely limits the large-scale application of natural spider silk in modern scientific and technological life. Furthermore, the biomimetic spider silk produced by existing technologies such as genetic engineering and biological engineering is used to manufacture biological materials, and synthetic silk materials, which has been proved to be far inferior to natural spider silk. Based on this, the team of the Yunnan Provincial Key Laboratory of Entomological Biopharmaceutical R&D has made breakthroughs in the key technology of large-scale cultivation of natural spider silk, through the cooperation of the laboratory and Hainan Spider King Biotechnology Co., Ltd. and other firms, hundreds of kilograms of natural spider silk can be mass-produced each year, thus breaking the bottleneck of the new industrial development of the new medical aesthetic material strategy in one fell swoop.

**Fig. 1:**
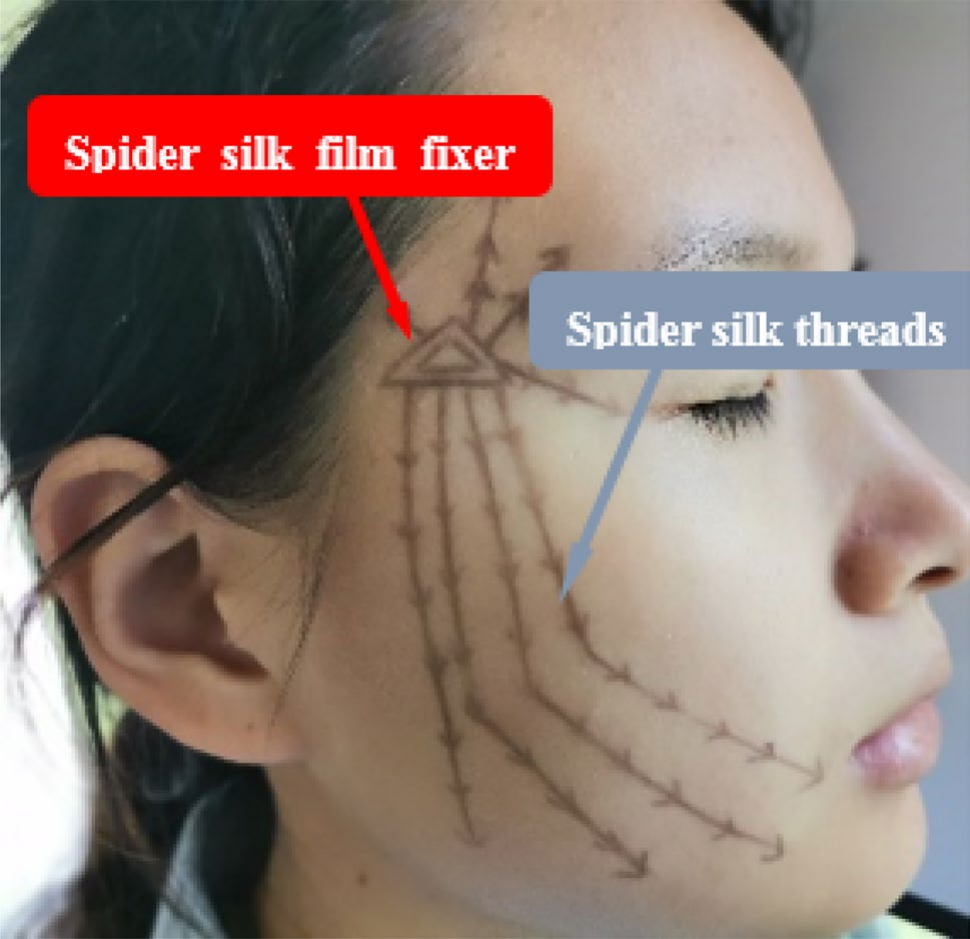
Illustration of spider silk sutures used in thread-lift

**Fig. 2:**
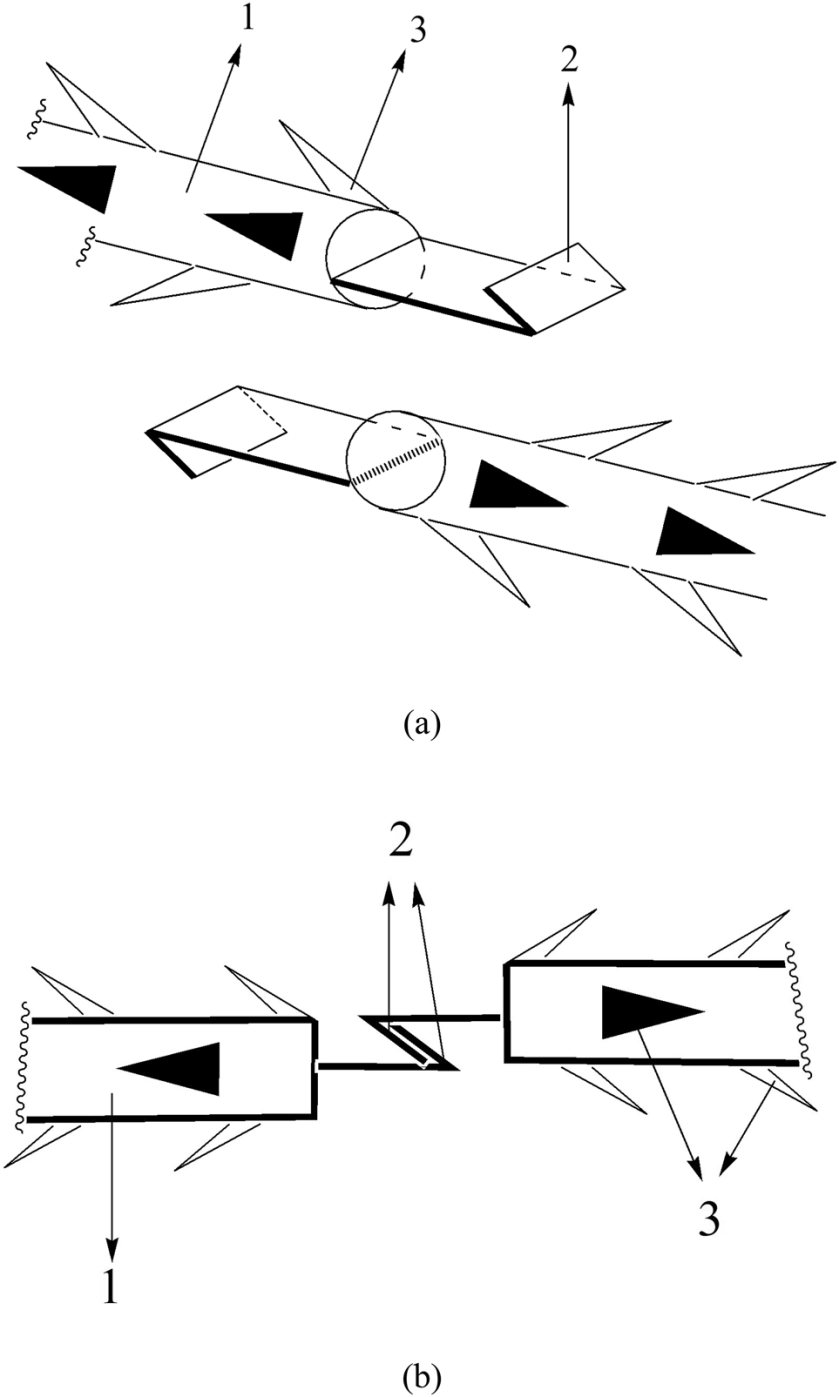
Illustration of the innovation of a flat-headed shackle-type hook biocompatible reverse-stretched barbed sutures. **a** front and rear view of sutures; **b** two barbed sutures connect by hook up with each other; **1** suture body; **2** flat-headed shackle-type hook; **3** the barbs of suture

**Fig. 3:**
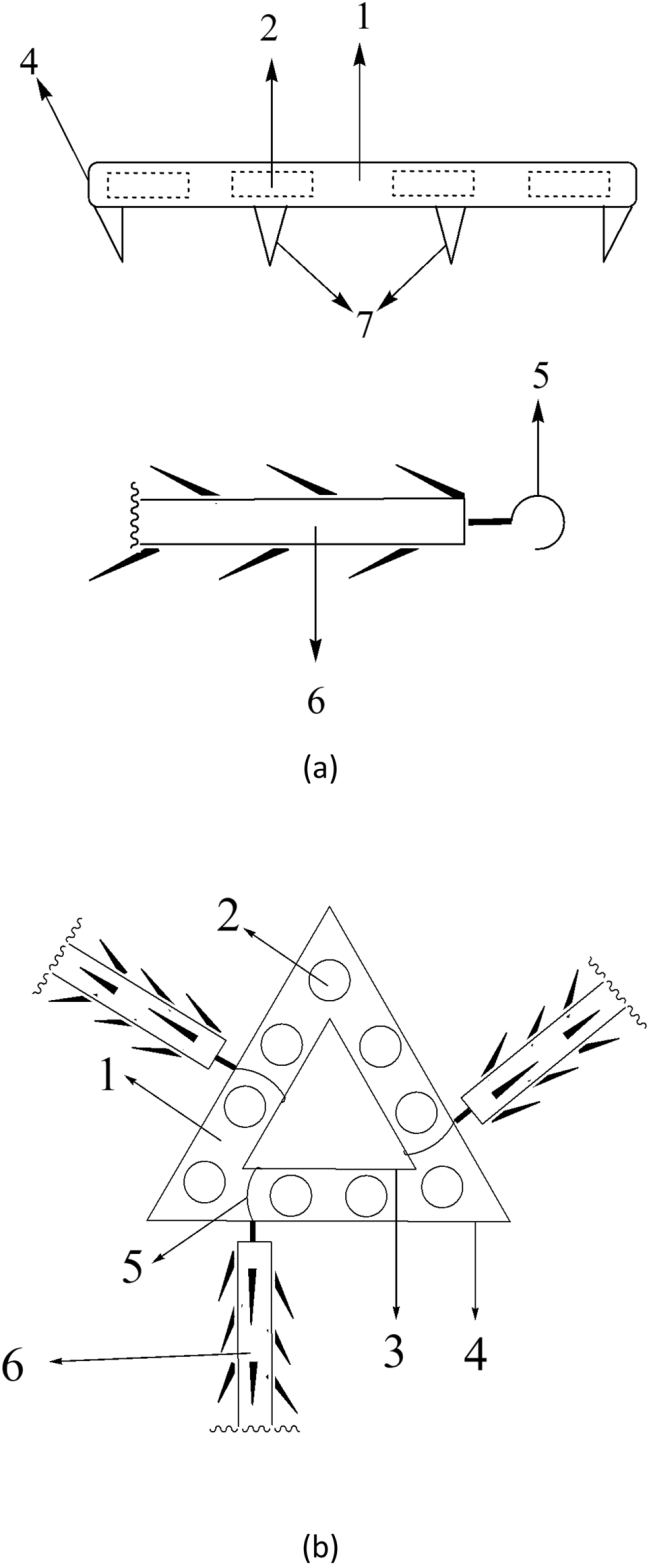
Illustration of the innovation of a triangular thin film fixer with a circular buckle holes prepared by natural spider silk. **a** vertical view of the device; **b** the application of the barbed sutures hooked on the thin film fixer; **1** main body of triangular thin film fixer; **2** circular buckle holes; **3** the inner edge of triangular thin film fixer; **4** the outer edge of triangular thin film fixer; **5** the hooks of suture; **6** the suture with barbs; **7** the fixing pins under the fixer

**Fig. 4:**
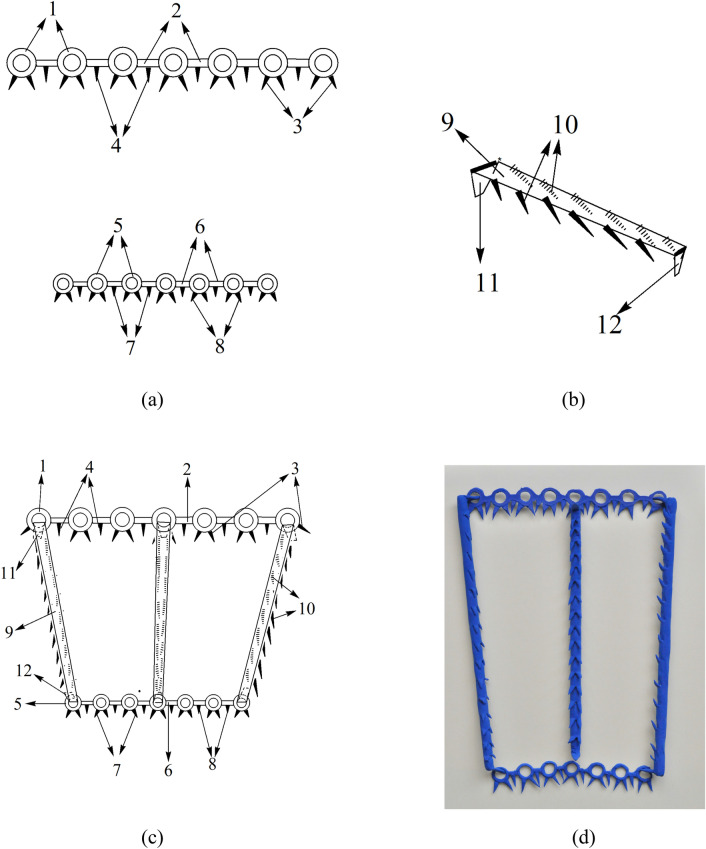
Illustration of the innovation of a barb-containing fixer with a large circular buckle hole and a small circular buckle hole, and the shovel-type buckle piece hook with barbs prepared by natural spider silk. **a** planform of circular buckle hole with barbs; **b** vertical and side view of shackle-type suture with barbs; **c** plastic body lift and hook fixer; **d** the rubber model of the device; **1, 5** circular buckle holes; **2, 6** connective band between circular buckle holes; **3, 7** spines on circular buckle hole; **4, 8** spines on connective band; **9** buckle connective pieces; **10** the barbs of the shovel-type buckle piece; **11, 12** the shovel-type buckle piece hooks

Now that the Laboratory overcomes the shackles of insufficient biocompatible materials, they established a guarantee system on natural spider silk resources, and then invent a composite preparation with natural spider silk combined with biodegradable polymer materials, thus offered the beauty barbed sutures and beauty barbed sutures suit device which gathered the “eight advantages” simultaneously (Fig. 1). Herewith listed a few examples made from spider silk: (1) a flat-headed shackle-type hook biocompatible reverse-stretched barbed sutures [59] (Fig. 2); (2) natural spider silk participates in the preparation of a barbed suture with groove type, which is composed of a suture thread body, a reserved buckle groove for hooking barbs, and lateral barbs on the suture thread [60]; (3) a circumferential thin film fixer with a circular buckle holes prepared by natural spider silk [61]; (4) a triangular thin film fixer with a circular buckle holes prepared by natural spider silk [62] (Fig. 3); (5) a circular thin film fixer with multiple stack types buckle holes prepared by natural spider silk [63]; (6) a barbed suture thread for a biocompatible beauty thread lifting with a hollow round buckle prepared by natural spider silk [64]; (7) a suture thread for a biocompatible beauty thread lifting with buckles and barbs alternated prepared by natural spider silk [65]; (8) a suture thread for a biocompatible beauty thread lifting with double hooks prepared by natural spider silk [66]; (9) a honeycomb-shaped beauty barbed sutures fixing device prepared by natural spider silk [67]; (10) in addition, the team also pioneered the creation of the breast-lifting and hip-lifting beauty barbed sutures materials containing spider silk. For example, natural spider silk is involved in the preparation of a biocompatible breast enhancement plastic body lift and pull hook fixer. The device is composed of a barb-containing fixer with a large circular buckle hole at the distal nipple end and a small circular buckle hole at the proximal nipple end, and several barbed and shovel-type buckle piece hook. The buckle piece hook can be inserted into the large and small round buckle hole of the plastic lifting suture hooking and fixing device to form a patented device together [68] (Fig. 4).

## Conclusion and Prospect

### Conclusion

Cosmetic thread lifting is proposed as a technology to achieve suspension and facial rejuvenation without surgery, which has won the interest between patients and surgeons. Although “thread-lifting plastic surgery” is increasingly popular, it cannot completely act as a substitute for open surgery, because of the data on its indications, complications, efficacy and long-lasting effect of results still need to be further observed and summarized. Nevertheless, with the advent of the so-called “lunch time” lifting technique, this technology is gaining popularity among white-collar customers [42]. Moreover, novel and different barbed threads are constantly being developed and entering into the market, and technological advancements may bring new industrial growth points and new application markets in further future. In order to better evaluate the efficacy, safety and optimal treatment options of thread lifting, the medical aesthetics market needs further multi-center research, including the types and number of sutures, larger samples, and longer follow-up time as well as the result evaluation [11].

The future thread-lifting technology should be used in the following aspects: (1) to ensure that new technologies in the industry can maintain continuous progress, future research on sutures should be considered in a double-blind manner for objective and standardized analysis of postoperative facial suspension at fixed intervals [25]; (2) researchers need to compare “suture suspension” with standard plastic surgery techniques for future coordinated investigation; (3) laboratory data and animal research should be performed to evaluate the biomechanical and biochemical responses of sutures in biological environments [27]; (4) the influence of thread lifting on the original face and the effect of repeated facial movements on it should be considered.

### Prospect

The thread lifting beauty sutures and set devices developed by the author team precisely resolve the key problem of the biocompatibility, rejection, flexibility, easy plasticity, aging performance, shape and usage characteristics of the thread-lift cosmetic suture, and self-absorbed by biological organisms, in this way, the “eight advantages” were gathered together innovatively by the creative general technology [59–68]. Therefore, although the above-mentioned products are still in the pre-clinical experiment, and complete laboratory animal and volunteer experiments are still being recruited and implemented, it is foreseeable that such a series of innovative products will have an enormous development potential and industrialization space in the future medical and aesthetic industry thread-lift materials market.

Yunnan Provincial Key Laboratory of Entomological Biopharmaceutical R&D has long been devoted to the research and development as well as market expansion of insect and arachnoid biomedicine. At present, it has formed a strategic emerging industry innovation R&D cluster with several pharmaceutical and cosmetic companies, focusing on natural spider silk related biomedical engineering materials and medicine. The research and development objects include more than 100 patented products such as cardiac stents, internal medicine, surgery, infection, burns, dentistry, urology, etc., which will be published in the form of patents and research papers in the coming.

The author is very willing to see numerous enterprises and universities as well as professional research institutes across the country jointly tackle the pivotal problems in such biocompatible medical materials that are in line with the forefront of future development strategies, can we gain sufficient progress in the industrial chain for the resource advantage of natural spider silk in China. The development of Arachnids biomedicine belongs to a strategic emerging industry. Only by pooling the strengths of the collectives to form a superior industrial chain and promote the establishment of industrial clusters, is the principal road for high-quality development of Chinese thread lifting beauty industry, and lead the industry in the forefront of the world.

## Postscript

The chief author (YZ) cherishes the memory of Prof. Zhou Jun deeply with this article. 25 years ago, Mr. Zhou used a heavy hammer to open up my scientific research ideas. After finishing the postdoc career at the Laboratory of academician Prof. Sun Handong at Kunming Institute of Botany, CAS, YZ has crossed the river by feeling the stones and explored different professional fields such as total synthesis, new drug development, pharmacology, pharmacy, and biotransformation; to meet the satisfaction and requirement of different industries. It is because of deep experience in different industries that YZ truly feels himself a shallow comprehensive foundation and a narrow knowledge. The span of his scientific career was quite large for several times, including the latest once, 10 years ago, YZ entered the insect biomedicine R&D region under the invitation of Dali University. In fact, they are all implementing Prof. Zhou’s legacy will: “Chinese young scientists should be determined to lead a new scientific research direction, better emerging sectors of strategic importance in the future, and do something that others have not done yet”. For 25 years, the old gentleman’s spur had been ringing in his ears, which made YZ deeply understand the verve of the prophet Mr. Chen Yinque when he was lecturing in Tsinghua University, “I don’t talk about what the predecessors have said, I don’t talk about what others have said, I don’t talk about what I have said before”. Only as a revolutionist of the old generation, will have such a charming character; just deserve to go up in such a natural and unrestrained style. YZ is always willing to follow the old gentleman’s teachings and implement his wishes. Prof. Zhou taught us to “don’t follow the usual path, don’t follow others, otherwise you will always be a mediocre person”, we think this is also one of the reasons why the old gentleman dared to stand up and angrily join the Communist Party during the Kuomintang rule, to transcend the worldly life and pursue the truth.

In view of the somewhat pompous atmosphere of Chinese science and academia in recent decades, it is of great benefit to remember the old man’s pursuit of origin and his courage to explore the “heterodoxy”. The thinking of “how to closely integrate Chinese medicine theory and phytopharmaceutical chemistry” that he advocated in the 1990s, now seems to be leading, pioneering thinking in domestic and foreign academic circles. Thus it can be seen that the old man had a profound academic foundation in China Pharmaceutical University, where he was graduated as a bachelor. The application of natural spider silk and bioengineering spider silk is a big industry with an annual output value of nearly 30 billion YUAN RMB. As for how to develop in the future, we are still ready to practice the will of the old man according to the words of Prof. Zhou: “Don’t follow, to lead”. I hope that our persistence in the spirit of freedom of scientific research will please the old gentleman who must be around Karl Marx in the Pure Land of the West, and he will be satisfied to see that his surviving students are still tirelessly and courageously implementing his advanced ideas and thoughts. Perhaps this belongs to the immortality of his spirit and the inheritance of his ideas.
